# Fluoroscopy-guided balloon blocking combined with thrombin injection for pseudoaneurysm of the common femoral artery

**DOI:** 10.1097/MD.0000000000046690

**Published:** 2025-12-26

**Authors:** Zhengli Liu, Yuqing Xia, Jie Kong, Jianping Gu, Meiqi Xia

**Affiliations:** a Department of Interventional Radiology, Nanjing First Hospital, Nanjing Medical University, Nanjing, Jiangsu, People’s Republic of China; b Department of Cardiovascular Medicine, Nanjing Gaochun people’s Hospital, Nanjing, Jiangsu, People’s Republic of China.

**Keywords:** balloon blocking, fluoroscopy, interventional therapy, pseudoaneurysm, thrombin

## Abstract

**Rationale:**

Common femoral artery pseudoaneurysm is a frequent complication of interventional puncture. Traditional compression has high failure rates and causes discomfort, while ultrasound-guided thrombin injection is limited in complex cases (e.g., multiple pseudoaneurysms). This report explores a new, effective solution.

**Patient concerns:**

A patient with multiple post-intervention pseudoaneurysms sought effective, minimally invasive treatment to avoid conventional therapy drawbacks.

**Diagnoses:**

Multiple common femoral artery pseudoaneurysms were diagnosed via clinical assessment and imaging after interventional puncture.

**Interventions:**

X-ray fluoroscopy-guided balloon blocking plus thrombin injection was performed to induce pseudoaneurysm lumen thrombosis.

**Outcomes:**

No treatment complications occurred; fluoroscopy enabled accurate occlusion visualization. Complete pseudoaneurysm occlusion was confirmed at 1-month follow-up.

**Lessons:**

This approach is viable for pseudoaneurysms refractory to compression or ultrasound-guided thrombin injection, serving as a useful supplement to traditional compression.

## 1. Introduction

Femoral artery pseudoaneurysm (FAP) is a relatively common vascular disease that is mainly caused by localized damage to the wall of the common femoral artery resulting in blood extravasation and the formation of a cystic structure that communicates with the lumen of the artery. This lesion usually occurs at the bifurcation of the common femoral artery, especially at the junction of the common femoral artery and superficial femoral artery.^[1]^ Common causes of common FAPs include trauma, surgery, infection, and atherosclerosis. Among them, medically derived pseudoaneurysms are more common in the clinic, and during puncture for interventional therapy, patients with atherosclerosis have weakened elasticity of the vessel wall, which is prone to injury. At the same time, if the puncture position is incorrect, improper anticoagulation and antiplatelet therapy, insufficient compression at the puncture point after the operation, and patients get out of bed too early, it will also increase the risk of pseudoaneurysm.^[2]^ FAP mostly cannot be healed by itself, and if it cannot be treated in time it can have the complications such as rupture of the aneurysm cavity and bleeding, compression of the surrounding tissues, and thrombus dislodgement in the cavity of an aneurysm, and so on. The main treatment modalities for pseudoaneurysms of the common femoral artery include manual local compression, overlay stent implantation, surgical incision repair, and ultrasound-guided tumor lumen thrombin injection (UGTI),^[3]^ but all of them have certain limitations. In our center, we reported a case of bilateral external iliac artery pseudoaneurysms and left common FAP after iliac artery angioplasty, for which the patient with left common femoral artery aneurysm received X-fluoro guided balloon blocking combined with thrombin injection. We found that X fluoroscopy-guided balloon blocking combined with thrombin injection can show the degree of occlusion of the pseudoaneurysm more intuitively and precisely, and provide clinical reference value.

## 2. Case presentation

**Figure 1.**
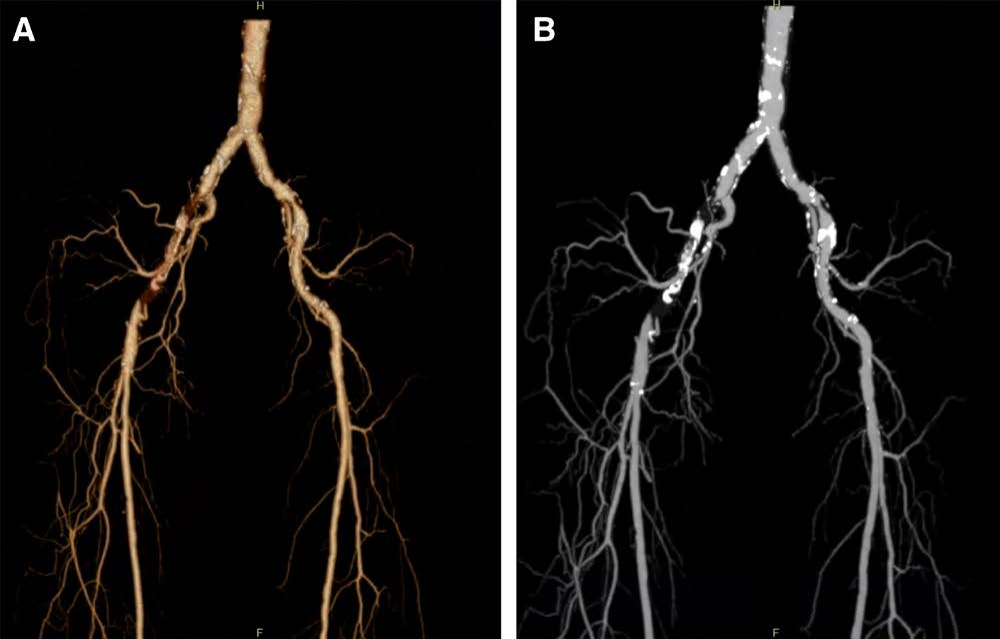
(A) Patient’s local CTA suggests right iliac artery with thrombosis, with no signs of pseudoaneurysm. (B) Maximal intensity projection shows heavily calcified plaque formation. CTA = computed tomography angiography.

The patient is a 58-year-old male, who was admitted to the hospital for “intermittent claudication of the right lower limb for more than 1 month.” The patient’s treatment process and data were agreed by close relatives, and a written informed consent was signed. The patient has a long history of diabetes and hypertension with poor glycemic control. The patient had Stage 2 according to Rutherford classification. Computed tomography angiography of the lower limb arteries performed at the local hospital showed signs of atherosclerosis and severe calcification with local truncation of the distal abdominal aorta, bilateral iliac arteries, and bilateral lower limb arteries (Fig. 1). Our intraoperative arteriography revealed a filling defect within the patient’s right iliac artery, but also a stenosis of the iliac artery. Combined with the patient’s elevated D-dimer(4.34 μg/ml at hospital admission), we considered stenosis complicating subacute arterial thrombosis, and thrombolysis of the right external iliac artery was performed. After the operation, the patient was treated with urokinase transcatheter thrombolysis. After thrombolysis, the patient’s thrombus was dissolved, but the right external iliac artery still had severe stenosis. After thrombolytic therapy with urokinase cannulation, we implanted a self-expandable stent because the patient still had residual severe stenosis of the right iliac artery. Balloon dilatation of both iliac arteries were also performed. Postoperative follow-up imaging showed that the blood flow of bilateral external iliac arteries was smooth, and there was no sign of pseudoaneurysm. After removing the sheath, the puncture point was bandaged with localized pressure, and the left lower extremity was braked for 6 hours after surgery, and the patient got out of bed after 24 hours, and the bandage was lifted on the third postoperative day without any obvious abnormality. The patient was discharged with postoperative skin temperature of the right lower limb, disappearance of intermittent claudication symptoms, and Rutherford Stage 0.

The patient was hospitalized in our department for review after 1 month of routine treatment. We palpated the femoral artery puncture site and found a localized suspicious pulsatile mass with pressure pain, suspecting a FAP. A complete computed tomography angiography scan of the lower extremity arteries revealed bilateral external iliac artery pseudoaneurysms and left common FAPs, with limited hematoma formation at the bilateral external iliac artery pseudoaneurysms. The neck width of the right iliac artery aneurysm was 2.15 mm, and the size of the aneurysm was 1.96 × 1.54 cm; the neck width of the left iliac artery aneurysm was 2.46 mm, and the size of the aneurysm was 1.69 × 1.34 cm; and the neck width of the left common femoral artery aneurysm was 3.13 mm, and the size of the aneurysm was 1.05 × 1.04 cm. At the same time, the patient suffered from multiple calcified arteries in the lower limbs on both sides.

After clarifying the site of the patient’s pseudoaneurysm, we decided to perform an intervention for the patient. The study was approved by the ethics committee of our hospital (KY20241014-02). The patient was sterilized and sheeted bilaterally in the groin area, and to minimize vascular injury, the right superficial femoral artery was punctured using an 18G minimally invasive puncture needle with a 4F minimally invasive puncture sheath. We introduced a pigtail catheter with the cephalic end placed in the abdominal aorta and injected contrast for bilateral lower extremity arteriography. The imaging showed bilateral external iliac artery pseudoaneurysm formation and left common FAP formation. We then replaced the 7F introducer sheath (COOK) with the head end of the sheath to the left common iliac artery and used a guidewire with a catheter to pass through the left iliac artery with the head end of the guidewire placed in the left superficial femoral artery. We first localized the left external iliac artery aneurysm under Roadmap guidance, introduced a 9mm × 5cm covered stent (VIABAHN® Endoprosthesis with Heparin Bioactive Surface, GORE®) anchored to the left external iliac artery aneurysm, and replaced the stent with an 8-mm × 6-cm balloon dilatation catheter (Abbott) for in-stent posterior dilatation. The Amplatz guidewire was then retained, and a 9mm × 5mm covered stent was anchored to the right external iliac artery aneurysm in the same way. Then we performed post-balloon dilatation. The head end of the catheter was placed in the abdominal aortogram for review, which showed that bilateral external iliac artery aneurysms were not visualized.

We then performed left common femoral artery angiography using the capabilities of 3D DSA to confirm the location of the left common FAP. The tip of the guidewire was again placed in the left superficial femoral artery, and Roadmap guidance was used to seal the pseudoaneurysm rupture using an 8-mm × 6-cm balloon, which was located in the center of the balloon, and to confirm that the pseudoaneurysm was not visualized by using a sheath cephalad angiogram. We removed the pressure from the balloon and positioned the left common FAP for puncture under fluoroscopic guidance. The solution for injection into the aneurysm cavity was prepared in advance (500 U of thrombin + 1 ml of contrast + 1 ml of saline mixed thoroughly), and a minimally invasive puncture needle was used to puncture into the aneurysm cavity to visualize the pulsatile arterial blood ejection, and contrast was injected to visualize the aneurysm cavity and to confirm that the tip of the needle was located in the aneurysm cavity. The balloon was then filled using a pressure filling system to block the common femoral artery flow, and the pressure was maintained at 8 atm. A thrombin + contrast mixture was injected into the pseudoaneurysm cavity through the retention puncture needle, and the pseudoaneurysm cavity was visualized completely under fluoroscopy without spillage of the contrast agent. The pressure of 8 atm was maintained for 3 minutes, then we withdrew the balloon, and 1 minute later, left lower extremity arteriography was performed through the sheath, and the left common FAP lumen was not visualized, and the distal endings were well visualized. During the aneurysm injection process, the patient’s vital signs were paid attention to, especially the temperature and function of the distal end of the lower extremity artery. During the treatment, the patient had no obvious complaints of discomfort, and the peripheral blood supply was good. After the operation, the local puncture point was bandaged with pressure, and the patient returned to the ward.

**Figure 2.**
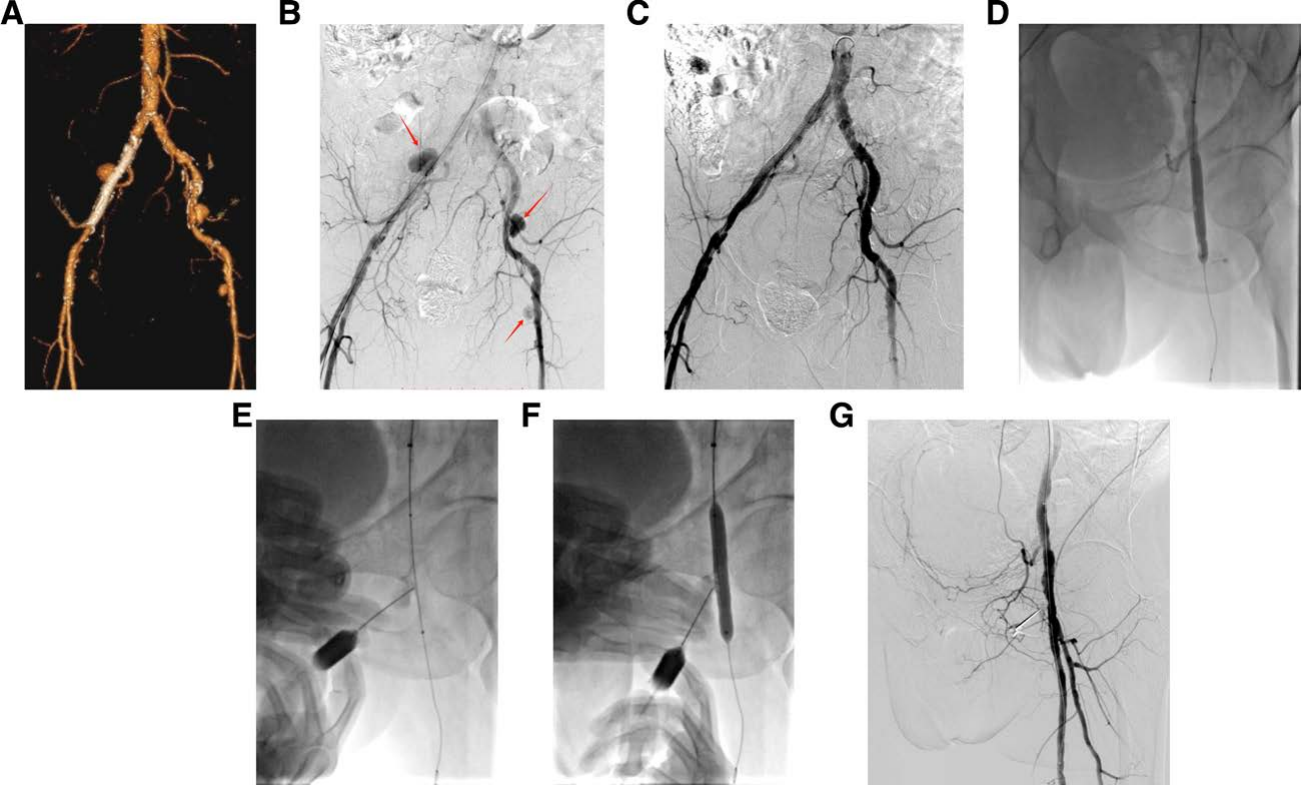
(A) Preoperative CTA showed that the patient’s bilateral external iliac arteries and left common femoral artery pseudoaneurysms were formed, and atherosclerosis was obvious; (B) We performed imaging in the abdominal aorta, confirming the location and morphology of the 3 pseudoaneurysms; (C) We first dealt with bilateral external iliac artery pseudoaneurysms, and bilaterally implanted a 9 mm × 5 cm overlying stent (Viabhan, GORE) anchored to the aneurysmal rupture. The contrast review suggested that bilateral external iliac artery aneurysms were not visualized; (D) after using a balloon to seal the neck of the left FAP aneurysm, the FAP was not visualized by overhead manual push contrast; (E) the balloon was withdrawn, the left FAP was punctured, and contrast was injected to confirm that the tip of the needle was in the FAP; (F) the balloon was filled and sealed the neck of the FAP aneurysm, and thrombin + contrast were injected to make the aneurysm cavity visualized, and the sealing was maintained for 3 minutes; (G) contrast review did not show the left FAP visualization. CTA = computed tomography angiography, FAP = femoral artery pseudoaneurysm.

Postoperative cardiac monitoring was performed to observe the peripheral blood supply, and no obvious abnormal signs were seen. The patient was given a localized pressure bandage in the left inguinal area after the operation, and the patient was given an extension brake for 6h on the puncture side, and the patient should not bear weight on the lower limb on the puncture side for 1 day. Bedside ultrasonography on the third postoperative day showed thrombosis in the left common femoral artery aneurysm, and no obvious blood flow signal was seen. The patient was discharged from the hospital and continued antiplatelet and anticoagulation therapy after discharge. The specific treatment regimen was aspirin 100 mg once daily, rivaroxaban 2.5 mg twice daily, and atorvastatin 20 mg once nightly. The patient did not complain of obvious discomfort after operation, and was discharged after evaluation. Reevaluation by ultrasound was performed 1 month after discharge and no FAP was seen in the left inguinal region. The patient is still in follow-up and insists on oral medication. Six months after discharge in this patient, no recurrence of pseudoaneurysm was found in the local ultrasound examination. The patient’s typical course of treatment is shown in Figure 2.

## 3. Discussion

A pseudoaneurysm is a rupture of an artery that causes blood to flow out into the surrounding tissue space, where some of the blood is encapsulated by the tissue and forms a hematoma. The puncture route of the femoral artery is the most common access in interventional procedures and FAP is the most common complication during interventional puncture with an incidence of up to 2.9%.^[4]^ Among them, patients’ advanced age, male gender, hypertension, diabetes mellitus, and obesity are independent risk factors for FAP, which may be related to patients’ poor vascular conditions. It has also been shown that a sheath size > 6F is independently associated with an increased risk of FAP,^[5–7]^ where blood passes through the arterial rupture into the extravascular saccular lumen and travels back and forth to form a pulsatile cystic hematoma. In this case, the patient had a previous history of hypertension and diabetes, extensive arteriosclerosis, and severe calcified plaques in bilateral iliac arteries and superficial femoral arteries. Intraoperative balloon dilatation of the iliac artery may cause tearing of the arterial intima. The patient failed to straighten and brake effectively after surgery, which is also the reason for the pseudoaneurysm in the later stage of the patient.

Conventional ultrasound-guided compression of pseudoaneurysms is effective has a low complication rate and is widely used in clinical practice. The advantage of ultrasound guidance is to avoid ionizing radiation, in addition to avoiding the risk of contrast induced nephropathy. However, for physicians, ultrasound-guided compression of pseudoaneurysms is time-consuming and requires a constant force for a long period.^[8]^ At the same time, the patient will have a longer period of pain during compression, and the affected limb needs to be braked and compressed for a long period after compression, and the local puncture point needs to be more complicatedly bandaged at a later stage.^[9]^ If the compression strength is not uniform in the later stage, aneurysm recurrence may occur. Ultrasound-guided thrombin Injection for the treatment of 1 to 6.5 cm FAPs is also currently used as a first-line treatment,^[10]^ and the complications of UGTI are uncommon, with a reported embolization rate of 0.8% and a risk of venous thrombosis of 0.2%,^[11]^ but this method is more demanding for the operator himself and the ultrasound-guided compression is not exact, and it is prone to complications such as ectopic embolization if poor compression is performed. At the same time, for pseudoaneurysms with outflow tract, ultrasound-guided thrombin injection will increase the risk of thrombin ectopic embolization. Angiography can accurately determine the outflow tract of pseudoaneurysm. Meanwhile, overlay stent implantation can also cover pseudoaneurysm ruptures, but it is economically costly and more complicated to perform, and stent release requires prolonged practice.

In this study, the use of a balloon to seal the pseudoaneurysm rupture and inject thrombin into the lumen of the aneurysm has the following advantages: 1. The whole injection process can be carried out under fluoroscopy, which is more intuitive, and avoids the possibility of ectopic embolization of the lower limb arteries caused by the spillage of thrombin.2. For obese patients with pseudoaneurysms, the adipose tissue greatly attenuates the ultrasound energy, which leads to a decrease in beam intensity, deterioration of image quality, and a decrease in the accuracy of assessment of the vascular structure. Assessment is less accurate.3. X-ray fluoroscopy-guided sealing of the aneurysm rupture using a balloon followed by high-grade aneurysms can be performed with overlying stent implantation. In this case, the patient had multiple aneurysms and was able to treat all aneurysms with a single puncture.

The advantages of this new pseudoaneurysm treatment modality compared to other pseudoaneurysm treatment modalities presented herein are mainly the visualization of the entire procedure and the ability to see the extent to which thrombin is diffused within the aneurysm cavity during injection. We suggest that this approach can be considered in the following cases: patients with multiple aneurysms requiring re-intervention or imaging review can use this approach for endoluminal operation; patients with pseudoaneurysms that still exist after conventional compression or aneurysms that still cannot be closed after ultrasound-guided thrombin injection can use this treatment approach. However, this interventional approach still has some limitations. First, this approach needs to be done under fluoroscopic guidance again and requires a procedure under fluoroscopy. This approach requires re-puncture, which is to some extent not as simple and easy as ultrasound-guided compression. When puncturing the contralateral artery, care should be taken to protect the target vessel and avoid damage to the contralateral artery as much as possible. This study has the following limitations. First, it is a single-case report with a small sample size and limited follow-up, so the generalizability of conclusions and long-term safety remain unvalidated. Second, the procedure relies on fluoroscopic equipment and secondary puncture, which increases radiation exposure for patients and operators, raises operational costs and vascular injury risks.

## 4. Conclusion

In conclusion, fluoroscopy-guided balloon blocking combined with thrombin injection is an effective method for the treatment of pseudoaneurysms and can be used as a supplement to other treatment modalities.

## Author contributions

**Conceptualization:** Zhengli Liu, Yuqing Xia, Meiqi Xia.

**Data curation:** Zhengli Liu, Yuqing Xia, Jie Kong, Meiqi Xia.

**Formal analysis:** Zhengli Liu, Jie Kong, Meiqi Xia.

**Funding acquisition:** Zhengli Liu, Yuqing Xia, Jianping Gu.

**Investigation:** Zhengli Liu.

**Methodology:** Yuqing Xia, Jianping Gu.

**Resources:** Zhengli Liu, Meiqi Xia.

**Software:** Zhengli Liu, Yuqing Xia.

**Supervision:** Jianping Gu.

**Validation:** Zhengli Liu, Jie Kong.

**Visualization:** Zhengli Liu.

**Writing – original draft:** Zhengli Liu, Yuqing Xia, Jie Kong, Jianping Gu, Meiqi Xia.

**Writing – review & editing:** Zhengli Liu, Jie Kong, Meiqi Xia.

## References

[1] Kurzawski J Janion-Sadowska A Zandecki L Sadowski M. Comparison of the efficacy and safety of two dosing protocols for ultrasound guided thrombin injection in patients with iatrogenic femoral pseudoaneurysms. Eur J Vasc Endovasc Surg. 2020;59:1019–25. 32014339 10.1016/j.ejvs.2020.01.009

[2] Naddaf A Williams S Hasanadka R Hood DB Hodgson KJ. Predictors of groin access pseudoaneurysm complication: a 10-year institutional experience. Vasc Endovascular Surg. 2020;54:42–6. 31578127 10.1177/1538574419879568

[3] Zhang Y Li T Liao CJ. Vascular surgical management of iatrogenic femoral artery pseudoaneurysm. Chin J Gen Surg. 2020;35:75–6.

[4] Hirano Y Ikuta S Uehara H. Diagnosis of vascular complications at the puncture site after cardiac catheterization. J Cardiol. 2004;43:259–65. 15242075

[5] Ates M Sahin S Konuralp C. Evaluation of risk factors associated with femoral pseudoaneurysms after cardiac catheterization. J Vasc Surg. 2006;43:520–4. 16520166 10.1016/j.jvs.2005.11.009

[6] Uppot RN Sahani DV Hahn PF Kalra MK Saini SS Mueller PR. Effect of obesity on image quality: fifteen year longitudinal study for evaluation of dictated radiology reports. Radiology. 2006;240:435–9. 16801372 10.1148/radiol.2402051110

[7] Wiseman JT Fernandes-Taylor S Barnes ML. Predictors of surgical site infection after hospital discharge in patients undergoing major vascular surgery. J Vasc Surg. 2015;62:1023–31.e5. 26143662 10.1016/j.jvs.2015.04.453 PMC4586313

[8] Dean SM Olin JW Piedmonte M Grubb M Young JR. Ultrasound-guided compression closure of postcatheterization pseudoaneurysms during concurrent anticoagulation: a review of seventy-seven patients. J Vasc Surg. 1996;23:28–34, discussion 34-5. 8558739 10.1016/s0741-5214(05)80032-1

[9] Franco CD Goldsmith J Veith FJ Calligaro KD Gupta SK Wengerter KR. Management of arterial injuries produced by percutaneous femoral procedures. Surgery. 1993;113:419–25. 8456398

[10] Yoo T Starr JE Go MR Vaccaro PS Satiani B Haurani MJ. Ultrasound-guided thrombin injection is a safe and effective treatment for femoral artery pseudoaneurysm in the morbidly obese. Vasc Endovascular Surg. 2017;51:368–72. 28560886 10.1177/1538574417708727 PMC5913734

[11] Morgan R Belli AM. Current treatment methods for postcatheterization pseudoaneurysms. J Vasc Interv Radiol. 2003;14:697–710. 12817037 10.1097/01.rvi.0000071089.76348.6a

